# Supplementary material for: Palustrine forested wetland vegetation communities change across an elevation gradient, Washington State, USA
Source: PeerJ. 2020 Apr 1;8:e8903. doi: 10.7717/peerj.8903 (PMC7127484; doi:10.7717/peerj.8903)
Supplement: Supplemental Information 1 [file peerj-08-8903-s001.docx]

Table S1.1. PERMANOVA test results for overstory composition by wetland zone. PERMANOVA results from 10,000 permutations constrained by block (plot location).

**A. Overstory**

| Factor | DF | SS | MSS | F | R^2^ | P |
| --- | --- | --- | --- | --- | --- | --- |
| Zone | 2 | 1.4118 | 0.70588 | 4.3362 | 0.22425 | **0.0005** |
| Residuals | 30 | 4.8836 | 0.16279 |  | 0.77575 |  |
| Total | 32 | 6.2954 |  |  | 1.0000 |  |

Between zone overstory PERMANOVA comparisons:

| **Treatment** | Buffer | Upper | Lower |
| --- | --- | --- | --- |
| Buffer | -- | F = 6.8211  R^2^ = 0.23054 | F = 5.9924  R^2^ = 0.23054 |
| Upper | *P* = 0.0002 | -- | F = 0.32079  R^2^ = 0.01579 |
| Lower | *P* = 0.0001 | *P* = 0.8182 | -- |

Table S1.2. PERMANOVA test results for overstory composition by wetland zone. PERMANOVA results from 10,000 permutations constrained by block (plot location).

**B. Understory**

| Factor | DF | SS | MSS | F | R^2^ | P |
| --- | --- | --- | --- | --- | --- | --- |
| Zone | 2 | 17.720 | 8.8599 | 30.07 | 0.17324 | **0.0001** |
| Residuals | 287 | 84.562 | 0.2946 |  | 0.82676 |  |
| Total | 289 | 102.282 |  |  | 1.0000 |  |

Between zone understory PERMANOVA comparisons:

| **Treatment** | Buffer | Upper | Lower |
| --- | --- | --- | --- |
| Buffer | -- | F = 38.967  R^2^ = 0.16444 | F = 46.491  R^2^ = 0.19826 |
| Upper | *P* = 0.0001 | -- | F = 2.791  R^2^ = 0.01463 |
| Lower | *P* = 0.0001 | *P* = 0.008 | -- |
