# Supplementary material for: Palustrine forested wetland vegetation communities change across an elevation gradient, Washington State, USA
Source: PeerJ. 2020 Apr 1;8:e8903. doi: 10.7717/peerj.8903 (PMC7127484; doi:10.7717/peerj.8903)
Supplement: Supplemental Information 2 [file peerj-08-8903-s002.docx]

Table S2. Correlations between individual species and NMDS ordination solutions.

Table S2.1 Overstory – Multiple regression correlations between individual tree species and the final ordination solution’s axes. Species codes correspond to those in Appendix three

| SPECIES | NMDS1 | NMDS2 | NMDS3 | R^2^ | P |
| --- | --- | --- | --- | --- | --- |
| ACCI | -0.0989 | -0.87794 | -0.46845 | 0.6061 | 0.001 |
| ACMA | -0.03457 | -0.78748 | -0.61537 | 0.3234 | 0.045 |
| ALRU | -0.23009 | 0.33748 | -0.91278 | 0.767 | 0.001 |
| COCO | -0.17577 | -0.97738 | -0.11757 | 0.1732 | 0.155 |
| FRLA | 0.93004 | 0.12148 | 0.34681 | 0.9509 | 0.001 |
| PSME | -0.15053 | -0.96084 | -0.23267 | 0.8801 | 0.001 |
| THPL | -0.66269 | 0.2781 | 0.69534 | 0.9229 | 0.001 |
| ILAQ | 0.04519 | 0.10724 | -0.99321 | 0.2634 | 0.09 |
| PREM | -0.16157 | 0.90435 | -0.39503 | 0.2807 | 0.028 |
| RUSP | 0.04519 | 0.10724 | -0.99321 | 0.2634 | 0.09 |
| COSE | -0.15441 | 0.42532 | -0.89177 | 0.3806 | 0.009 |
| TSHE | -0.24273 | 0.10065 | 0.96486 | 0.2425 | 0.058 |
| HODI | 0.29519 | -0.40389 | -0.86587 | 0.0529 | 0.616 |
| SPDO | -0.21864 | 0.70958 | 0.66985 | 0.0639 | 0.576 |
| RHPU | 0.21422 | 0.86833 | -0.44734 | 0.1336 | 0.212 |
| ABGR | -0.26926 | -0.47317 | 0.83882 | 0.0492 | 0.679 |
| TABR | -0.38152 | -0.14446 | 0.913 | 0.146 | 0.149 |
| OECE | 0.34663 | 0.68875 | -0.63677 | 0.0744 | 0.511 |
| PHCA | 0.34663 | 0.68875 | -0.63677 | 0.0744 | 0.511 |

Table S2.2 Understory – Multiple regression correlations between individual understory plant species and the final ordination solution’s axes.

| SPECIES | NMDS1 | NMDS2 | NMDS3 | R^2^ | P |
| --- | --- | --- | --- | --- | --- |
| ACCI | -0.3209 | 0.73261 | -0.60026 | 0.0576 | 0.001 |
| ACMA | -0.25142 | 0.67584 | 0.69284 | 0.0084 | 0.495 |
| ALRU | 0.55559 | -0.3912 | -0.73367 | 0.0099 | 0.44 |
| AMAL | 0.51914 | 0.76143 | 0.38824 | 0.0192 | 0.148 |
| ATFI | 0.76729 | -0.63201 | 0.10877 | 0.0141 | 0.233 |
| BENE | -0.90209 | 0.07274 | 0.42538 | 0.1446 | 0.001 |
| BLSP | -0.05696 | -0.49462 | 0.86724 | 0.0154 | 0.206 |
| BOMU | 0.12964 | 0.97845 | -0.16071 | 0.0186 | 0.159 |
| CAOB | 0.96162 | -0.11412 | 0.24954 | 0.481 | 0.001 |
| CADE | -0.06419 | -0.92832 | -0.3662 | 0.025 | 0.067 |
| CAHE | -0.54225 | 0.09042 | 0.83534 | 0.0149 | 0.212 |
| CASE | -0.31048 | 0.6598 | -0.6843 | 0.0502 | 0.002 |
| CLSI | 0.76063 | 0.15456 | 0.63052 | 0.0079 | 0.505 |
| COCO | -0.13396 | -0.89358 | 0.42844 | 0.0067 | 0.62 |
| COSE | 0.62315 | 0.47244 | -0.62328 | 0.1164 | 0.001 |
| DIPU | -0.01685 | -0.89718 | 0.44135 | 0.0165 | 0.178 |
| ELGL | -0.84249 | -0.51873 | -0.14536 | 0.0124 | 0.315 |
| EPAN | 0.08168 | 0.36527 | -0.92731 | 0.0022 | 0.969 |
| EQAR | -0.26546 | 0.79217 | -0.54954 | 0.0174 | 0.156 |
| FRLA | 0.25528 | -0.92536 | -0.28026 | 0.0807 | 0.001 |
| GAOV | -0.53909 | 0.77342 | -0.33348 | 0.0601 | 0.001 |
| GASH | -0.34071 | 0.66881 | -0.66077 | 0.5527 | 0.001 |
| GATR | -0.31013 | 0.15646 | 0.93773 | 0.1375 | 0.001 |
| GERO | 0.1921 | 0.13794 | 0.97163 | 0.0496 | 0.005 |
| GLEL | 0.17131 | 0.83307 | -0.52597 | 0.0054 | 0.684 |
| LAMU | -0.29967 | -0.03894 | 0.95325 | 0.0743 | 0.001 |
| LOIN | 0.58727 | 0.06024 | 0.80715 | 0.0337 | 0.026 |
| LUPA | -0.60126 | -0.23845 | 0.76264 | 0.0156 | 0.198 |
| MADI | -0.09517 | -0.2613 | 0.96055 | 0.001 | 0.971 |
| MOUN | 0.25412 | -0.07585 | 0.96419 | 0.0215 | 0.084 |
| OECE | 0.5885 | 0.35066 | -0.72849 | 0.0068 | 0.607 |
| PEPA | 0.20931 | -0.04255 | 0.97692 | 0.041 | 0.008 |
| PHCA | 0.52698 | -0.82713 | 0.19533 | 0.026 | 0.056 |
| POGL | 0.27343 | 0.02952 | 0.96144 | 0.0348 | 0.015 |
| POMU | -0.79075 | -0.40871 | 0.45571 | 0.4891 | 0.001 |
| PREM | 0.9793 | 0.20219 | 0.00971 | 0.0389 | 0.01 |
| PSME | -0.66588 | -0.74595 | 0.01278 | 0.0085 | 0.452 |
| PTAQ | 0.2865 | 0.60701 | 0.74125 | 0.2378 | 0.001 |
| RHPU | -0.06377 | 0.25729 | 0.96423 | 0.0167 | 0.185 |
| RILA | -0.22525 | 0.35886 | 0.90581 | 0.0217 | 0.102 |
| ROGE | -0.59871 | -0.12075 | 0.79182 | 0.0198 | 0.123 |
| RONO | 0.42156 | -0.83713 | 0.34858 | 0.0254 | 0.053 |
| RUAR | -0.88583 | -0.29904 | -0.35479 | 0.0013 | 0.945 |
| RULE | 0.02496 | 0.47216 | 0.88116 | 0.0463 | 0.003 |
| RUSP | 0.44914 | 0.64232 | 0.62105 | 0.126 | 0.001 |
| RUUR | -0.17527 | 0.9716 | 0.15899 | 0.1024 | 0.001 |
| SARA | -0.23974 | -0.3111 | 0.91964 | 0.0198 | 0.129 |
| SMRA | -0.97323 | -0.04299 | -0.22579 | 0.0162 | 0.188 |
| SMST | 0.36817 | 0.19092 | 0.90994 | 0.0132 | 0.268 |
| SODU | 0.39323 | -0.87441 | -0.28422 | 0.0222 | 0.077 |
| SPDO | 0.54044 | 0.08786 | -0.83678 | 0.2063 | 0.001 |
| SYAL | 0.28224 | -0.90369 | 0.32199 | 0.0738 | 0.001 |
| TITR | -0.93038 | -0.36581 | 0.02392 | 0.0426 | 0.006 |
| THPL | 0.57126 | -0.80249 | 0.17227 | 0.0061 | 0.62 |
| TOME | 0.24872 | 0.1578 | 0.95563 | 0.0167 | 0.172 |
| TRCE | -0.32043 | 0.69586 | -0.64273 | 0.0356 | 0.013 |
| TROV | -0.95347 | 0.00882 | 0.30137 | 0.0383 | 0.017 |
| TSHE | -0.27614 | 0.85176 | 0.44526 | 0.0413 | 0.01 |
| VIPA | 0.16253 | -0.98083 | 0.10751 | 0.0403 | 0.013 |
| VAOV | -0.17588 | 0.97874 | -0.10553 | 0.031 | 0.026 |
| VAPA | -0.65963 | 0.31075 | -0.68434 | 0.0169 | 0.175 |
