# Supplementary material for: Palustrine forested wetland vegetation communities change across an elevation gradient, Washington State, USA
Source: PeerJ. 2020 Apr 1;8:e8903. doi: 10.7717/peerj.8903 (PMC7127484; doi:10.7717/peerj.8903)
Supplement: Supplemental Information 3 [file peerj-08-8903-s003.docx]

Table S3. Full overstory and understory Indicator Species Analysis results

Table S3.1. Full Dufrene-Legendre indicator species analysis results for overstory plots.

| **Species** | **Four-letter code** | **Zone** | **Indicator value** | **Prob.** | **Wetland indicator status**  **(Western Mountains Valleys and Coasts)** |
| --- | --- | --- | --- | --- | --- |
| *Abies grandis* | ABGR | WB | 30.15 | 0.305 | *FACU* |
| *Acer circinatum* | ACCI | WB | 48.40 | 0.27 | *FAC* |
| *Acer macrophyllum* | ACMA | WB | 30.15 | 0.42 | *FACU* |
| *Alnus rubra* | ALRU | UW – LW | 58.39 | 0.72 | *FAC* |
| *Cornus sericea* | COSE | UW – LW | 52.22 | 0.145 | *FACU* |
| *Corylus cornuta* | COCO | WB | 42.64 | 0.105 | *FACU* |
| *Frangula purshiana* | FRPU | UW – LW | 24.85 | 1 | *FACU* |
| *Fraxinus latifolia* | FRLA | UW – LW | 81.62 | 0.035 | *FACU* |
| *Holodiscus discolor* | HODI | WB | 20.78 | 1 | *FACU* |
| *Ilex aquifolium* | ILAQ | UW – LW | 21.32 | 1 | *FACW* |
| *Oemleria cerasiformis* | OECE | UW – LW | 21.32 | 1 | *FAC* |
| *Physocarpus capitatus* | PHCA | UW – LW | 21.32 | 1 | *FACU* |
| *Prunus virginiana* | PRVI | UW – LW | 63.26 | 0.125 | *FACW* |
| *Pseudotsuga menziesii* | PSME | WB | 93.95 | 0.005 | *FAC* |
| *Rubus spectabilis* | RUSP | UW – LW | 21.32 | 1 | *FACU* |
| *Spiraea douglasii* | SPDO | UW – LW | 21.32 | 1 | *FAC* |
| *Taxus brevifolia* | TABR | WB | 30.15 | 0.31 | *FACW* |
| *Thuja plicata* | THPL | WB | 75.88 | 0.095 | *FACU* |
| *Tsuga heterophylla* | TSHE | WB | 55.30 | 0.025 | *FACW* |

Table S3.2. Full multi-level pattern indicator species analysis for understory plots.

| **Species** | **Four-letter code** | **Wetland zones** | | | **Indicator value** | **Probability** | **Mean height above OHWM (Meters)** |
| --- | --- | --- | --- | --- | --- | --- | --- |
| *Acer circinatum* | ACCI | WB |  |  | 25.61 | 0.015 | 0.64 |
| *Acer macrophyllum* | ACMA | WB |  |  | 10.00 | 1 | -0.36 |
| *Alnus rubra* | ALRU | WB |  | LW | 12.57 | 0.535 | -0.40 |
| *Amelanchier alnifolia* | AMAL |  |  | LW | 18.26 | 0.045 | -0.17 |
| *Athyrium felix-femina* | ATFI |  | UW | LW | 14.02 | 0.61 | -0.11 |
| *Mahonia nervosa* | MANE | WB |  |  | 52.92 | 0.005 | 0.61 |
| *Blechnum spicant* | BLSP | WB |  | LW | 12.57 | 0.675 | -0.36 |
| *Botrychium multifidum* | BOMU |  |  | LW | 16.67 | 0.105 | -0.55 |
| *Carex obnupta* | CAOB |  | UW | LW | 86.88 | 0.005 | -0.47 |
| *Carex deweyana* | CADE |  |  | LW | 10.54 | 0.285 | -0.42 |
| *Carex hendersonii* | CAHE | WB |  |  | 10.00 | 1 | 0.09 |
| *Calystegia sepium* | CASE | WB |  |  | 17.32 | 0.085 | 1.30 |
| *Claytonia sibirica* | CLSI |  | UW | LW | 14.51 | 0.42 | -0.75 |
| *Corylus cornuta* | COCO | WB |  | LW | 14.51 | 0.42 | 0.12 |
| *Cornus sericea* | COSE |  |  | LW | 37.59 | 0.005 | -0.25 |
| *Digitalis purpurea* | DIPU |  |  | LW | 10.54 | 0.41 | -1.50 |
| *Elymus glaucus* | ELGL | WB |  |  | 14.14 | 0.245 | 0.48 |
| *Chamerion angustifolium* | EPAN |  | UW |  | 10.00 | 1 | -0.16 |
| *Equisetum arvense* | EQAR | WB |  |  | 10.00 | 1 | 0.70 |
| *Fraxinus latifolia* | FRLA | WB | UW | LW | 57.83 | NA | -0.25 |
| *Gaultheria ovatifolia* | GAOV | WB |  |  | 24.49 | 0.015 | 1.24 |
| *Gaultheria shallon* | GASH | WB | UW | LW | 54.77 | NA | 0.26 |
| *Galium triflorum* | GATR | WB | UW | LW | 38.51 | NA | -0.19 |
| *Geranium robertanium* | GERO |  | UW | LW | 17.48 | 0.215 | -0.10 |
| *Glyceria elata* | GLEL |  | UW | LW | 12.57 | 0.66 | -0.39 |
| *Lactuca muralis* | LAMU | WB | UW |  | 24.49 | 0.04 | 0.09 |
| *Lonicera involucrata* | LOIN | WB |  |  | 24.19 | 0.065 | -0.23 |
| *Luzula parviflora* | LUPA | WB | UW |  | 10.00 | 1 | 0.105 |
| *Matricaria discoidea* | MADI |  | UW | LW | 38.10 | 0.035 | -0.28 |
| *Moneses uniflora* | MOUN |  | UW |  | 10.00 | 1 | -0.63 |
| *Oemleria cerasiformis* | OECE |  | UW |  | 18.65 | 0.065 | -0.27 |
| *Petasites frigidus ssp. palmatus* | PEPA |  | UW |  | 20.00 | 0.04 | -0.53 |
| *Physocarpus capitatus* | PHCA |  |  | LW | 24.39 | 0.01 | -0.70 |
| *Polypodium glycorrhiza* | POGL |  | UW | LW | 21.55 | 0.36 | -0.38 |
| *Polystichum munitium* | POMU | WB |  |  | 74.74 | 0.005 | 0.30 |
| *Prunus emarginata* | PREM |  |  | LW | 33.51 | 0.005 | -0.19 |
| *Pseudotsuga menziesii* | PSME | WB |  |  | 10.00 | 1 | 0.22 |
| *Pteridium aquifolium* | PTAQ |  | UW | LW | 45.86 | 0.005 | -0.13 |
| *Frangula purshiana* | RHPU | WB | UW | LW | 18.57 | NA | 0.12 |
| *Ribes lacustre* | RILA | WB |  | LW | 14.51 | 0.405 | -0.45 |
| *Rosa gymnocarpa* | ROGE | WB |  |  | 14.14 | 0.335 | 0.24 |
| *Rosa nutkanna* | RONO |  |  | LW | 23.57 | 0.005 | -0.87 |
| *Rubus armeniacus* | RUAR | WB |  | LW | 10.26 | 0.75 | -0.40 |
| *Rubus leucodermis* | RULE | WB | UW | LW | 23.49 | NA | 0.03 |
| *Rubus spectabilis* | RUSP |  | UW | LW | 36.27 | 0.005 | -0.39 |
| *Rubus ursinus* | RUUR | WB |  | LW | 35.61 | 0.025 | -0.16 |
| *Sambucus racemosa* | SARA | WB | UW | LW | 11.74 | NA | 0.52 |
| *Smilacina racemosa* | SMRA | WB |  |  | 14.14 | 0.345 | 0.90 |
| *Smilacina stellata* | SMST | WB | UW | LW | 11.74 | NA | -0.21 |
| *Solanum dulcamara* | SODU |  |  | LW | 18.87 | 0.34 | -0.42 |
| *Spiraea douglasii* | SPDO |  | UW | LW | 42.98 | 0.005 | -0.50 |
| *Symphoricarpos albus* | SYAL |  |  | LW | 38.22 | 0.005 | -1.00 |
| *Tiarella trifoliata* | TITR | WB |  |  | 20.41 | 0.065 | -0.07 |
| *Thuja plicata* | THPL | WB | UW |  | 18.71 | 0.245 | 0.738 |
| *Tolmiea menziesii* | TOME |  | UW |  | 10.00 | 1 | -0.35 |
| *Trisetum cernuum* | TRCE | WB |  |  | 14.14 | 0.29 | 1.14 |
| *Trillium ovatum* | TROV | WB |  |  | 20.00 | 0.04 | 0.81 |
| *Tsuga heterophylla* | TSHE | WB | UW |  | 10.00 | 1 | 0.42 |
| *Vaccinium ovatum* | VAOV | WB |  | LW | 10.26 | 0.365 | 0.03 |
| *Vaccinium parvifolium* | VAPA | WB | UW |  | 17.18 | 0.435 | 0.224 |
| *Viola palustris* | VIPA |  |  | LW | 10.54 | 0.815 | -1.12 |
